# Implementation of the norwegian ‘Starting right’ child health service innovation: implementation adjustments, adoption, and acceptability

**DOI:** 10.1186/s12913-021-06096-x

**Published:** 2021-01-23

**Authors:** Thomas Westergren, Eirin Mølland, Kristin Haraldstad, Åshild Tellefsen Håland, Unni Mette Stamnes Köpp, Liv Fegran, Eirik Abildsnes

**Affiliations:** 1 Faculty of Health and Sports Sciences, University of Agder, P.O. Box 422, 4604 Kristiansand, Norway; 2 NORCE, Universitetsveien 19, 4630 Kristiansand, Norway; 3 Sørlandet Hospital, P.O. Box 416 Lundsiden, 4604 Kristiansand, Norway; 4 Kristiansand Municipality, P.O. Box 4, 4685 Nodeland, Norway

**Keywords:** Child health, School health, Public health nurses, Health service innovation, Evidence‐based practice, Decision support, Implementation, Core concepts

## Abstract

**Background:**

An increased and/or stable proportion of the child and adolescent population reports symptoms of impaired health, and the symptoms can be identified early. Therefore, structured child- and parent-reported outcome measures need to be implemented in child and school health services for decision support and identification of children at risk. We aimed to (a) qualitatively examine adjustments of active implementation from the pilot implementation of the Norwegian ‘Starting Right’ health service innovation including an online child health assessment tool and practical routines, and (b) measure practitioners´ adoption and parental acceptability.

**Methods:**

We used a mixed-methods design to qualitatively examine adjustments from working notes and meeting memoranda, and quantitatively assess adoption and acceptability from user rates provided by the systems log. Twenty-one child and school health nurses (CSHNs) from two child health centers participated in the implementation pilot of online health assessments in children aged 2-, 4- and 6-year. We used a deductive and narrative analysis approach using Fixsen et al.´s core implementation components to code and sort adjustments.

**Results:**

Core implementation components were adjusted throughout the pilot implementation. Researchers´ increased their availability in reciprocity with staff evaluation to integrate active implementation adjustments. We launched a project for improved data systems integration. The overall CSHNs adoption rate was satisfactory and higher in center A, where a medical secretary supported the nurses through the entire pilot phase, than in center B (96 vs. 55 %). Parental acceptability rate was overall high (77 %) with increased rates among parents of 6-year-old children (98 %) compared with younger ones (78–85 %), and in cases where both parents received the questionnaires.

**Conclusions:**

The ‘Starting Right’ health service innovation implementation was actively adjusted by integration of core implementation components mainly based on staff evaluation. The CSHNs adopted the innovation which was also acceptable to parents.

## Background

Nationally mandated and publicly funded child health services aiming to safeguard child development and identify children at risk of motor, social, emotional, behavioral, and cognitive dysfunction are common in Northern Europe [1]. However, the content of these health services varies greatly, and none meet World Health Organization screening criteria [1]. International evidence is also sparse concerning effect of preventive child healthcare on clinical endpoints [2]. Services often rely on experience-based assessments rather than structured and evidence-based screening, support, and surveillance systems [1]. In Norway, a contributing reason is that in child and school health centers, the electronic patient records do not support structured data storage formats or the use of validated child- or parent-reported instruments to assess children’s development, health, and well-being. Thus, such instruments are scarcely used to support clinical decision-making, as they must be handled by pen and paper, and calculated manually. Consequently, Norwegian health authorities lack an overview of how child- and parent-reported health problems develop, and children at risk might not be identified and supported into more healthy trajectories. Preventive child healthcare is hence not evaluated by clinical endpoints.

An increased and/or stable proportion of the child and adolescent population reports symptoms of poor health, with a preponderance of mental health problems [3–5]. Symptoms can be identified early [6, 7], and are related to parental health and socioeconomic disadvantages [7–10], which are known to be transmitted through generations [11]. Therefore, the need to comprehensively identify and approach families and children in need or at risk at an early stage is prominent. Interventions in early childhood are cost-effective with higher rates of return than most other investments [12], and are supported by the United Nations Sustainable Development Goals position paper [13]. The Norwegian Council on Social Inequalities in Health also supports identification and mapping of difficult childhood circumstances and early interventions [14].

Assessment of child health and development varies widely according to the methods used, the professionals who are responsible, and the settings of assessment in different countries [1]. In a systematic review, Lines et al. [15] reported that nurses worldwide working with children at risk at all levels of health services hesitate to act for fear of damaging the parent–professional healthcare worker relationship, and that they request enhanced decision support. Structured evidence-based assessment tools may provide such decision support, in addition to several opportunities and gains [16–21]. For instance, population-based screening of general mental health using the Strength and Difficulties Questionnaire (SDQ) has been implemented successfully in Scotland, and is reported to add valuable decision support and to identify more cases for interventions [20]. This has also been confirmed at a global level [21]. Universal screening and routine outcome monitoring can in addition improve earlier identification of child problems [16], create opportunities for evaluation of early interventions and preventive services [17], enhance user involvement and communication with families as well as interdisciplinary collaboration [18], and help services improve [19]. Hence, Norwegian child and school health centers may benefit from implementation of structured screening assessments.

Child and school health centers in Norway are organized within the municipal primary health care system. Child and school health nurses (CSHNs) have 14 scheduled appointments with each child and his/her family before the age of six [1, 22]. In schools, CSHNs have scheduled appointments with children at ages six and 13, in addition to vaccination, weight/height/growth surveillance, and collaboration with the family and school staff concerning the child’s health on demand [22].

### The `Starting Right´ health service innovation

**Fig. 1:**
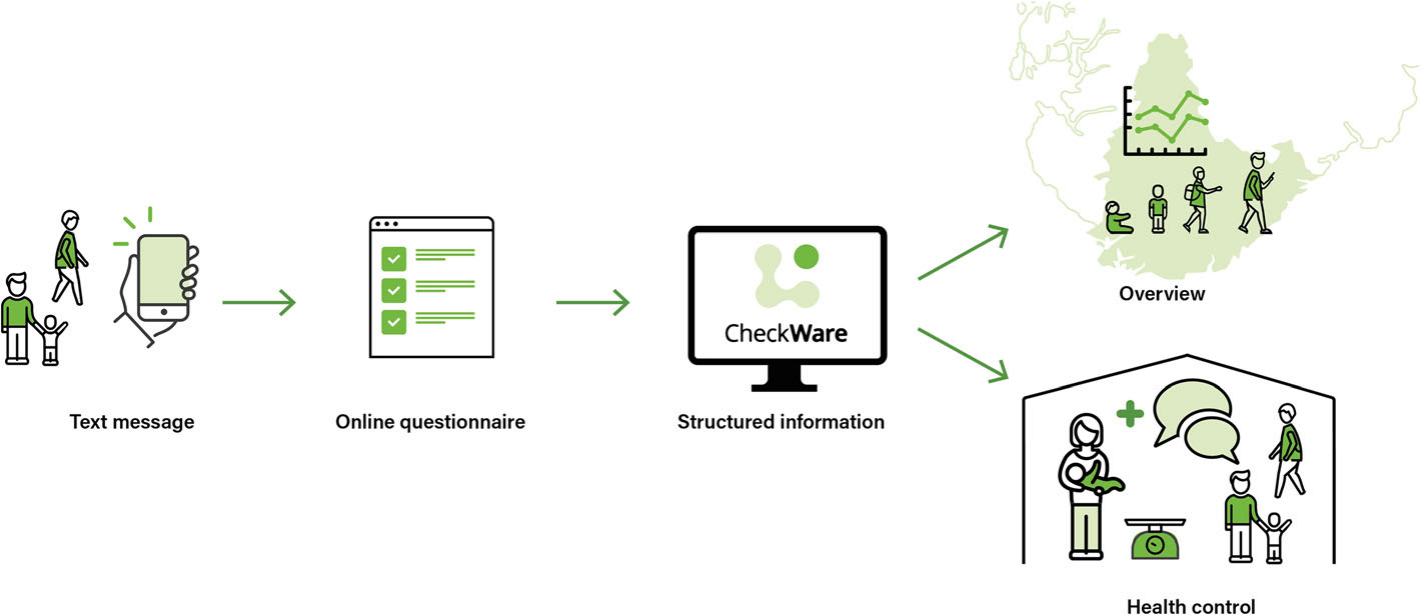
Data flow within the ‘Starting Right’ project. The online tool generates a report for decision support based on respondent-reported data, which can also be used for population health overview. The figure is created by Thomas Eikeland Fiskå at the University of Agder on request from the project, and published with permission

We initiated the `Starting Right´ health service innovation consisting of (a) a parent- and child-reported online structured health assessment tool developed by CheckWare Ltd. and (b) practical routines for use of the child health assessments in child and school health services among children aged 6 months to 16 years. We used well-validated questionnaires for general mental health (Strength & Difficulties Questionnaire (SDQ)) [23], health-related quality of life (KIDSCREEN-27) [24, 25], general development (Ages & Stages Questionnaire) [26], social-emotional development (Ages & Stages Questionnaire: Social–Emotional) [27], and anxiety (Spence Child Anxiety Scale, Short) [28]. In addition, we developed a simple form for the CSHN to plot height/weight and level of follow-up (0–4 according to a national standard) for the child. Through the use of structured format data storage, the innovation can provide municipal and county health authorities with an overview about the health and well-being of their child population, as warranted in the Public Health Act [29]. The questionnaires can be distributed to parents through a text message using a validated high-security internet link and secure identification through the Norwegian public e-services login system (ID-porten). Routines were designed to distribute questionnaires 9 days prior to an appointment, with a reminder 6 days later in case of nonresponse. CSHNs were free to choose whether they would distribute questionnaires to one or both parents. Before the scheduled appointment, CSHNs could log into the system and read a summarized report concerning the child based on each questionnaire used. Figure 1 illustrates the data flow in the online solution, which with a similar structure could be adopted nationally in child and school health services and adapted with additional questionnaires and/or to different users/patients and services.

### Implementation preparations and arrangements

To pilot implementation of the innovation into two child health centers (center A and B), we established a project group in December 2018 consisting of dedicated CSHNs from each center, the head of services, and the researchers. Participants of the project group attended monthly meetings. At each center, one CSHN with an official mandate for professional quality improvement served as the main contact between the staff and the project group. All CSHNs received education about the project, the clinical instruments used (5 h; May 2019), and the online tool (3 h; October 2019). Prior to implementation, we developed a project website (www.godtbegynt.no) and a pamphlet providing families and CSHNs with information about the project. We also published online instruction handbooks and videos for CSHNs about the assessment instruments and how to use the online tool on the website. Two researchers (EM and TW) provided the CSHNs with support when needed. We provided e-mail addresses and telephone numbers for online support upon request from the CSHNs. The implementation was piloted between October 2019 and January 2020, and comprised appointments for children aged 2, 4, and 6 years including two parental questionnaires (SDQ and KIDSCREEN-27).

### Aims of the study

According to Fixsen et al. [30], the implementation of evidence-based practice is often inconsistent and ineffective. In human services, the challenge is to build evidence and quality into the daily practice of multiple collaborating practitioners. This does not happen passively by delivering novel solutions and knowledge: novel practitioner behavior is actively created and supported by core implementation components [30]. Thus, the aims of this study were to examine ;

1. how the core implementation components were adjusted for the `Starting Right´ health service innovation,
2. success with tool adoption among staff in child and school health centers, and.
3. success with tool acceptance among parents responding to health assessments.

## Methods

We used a mixed-methods design applying what Palinkas et al. [31] label as a function of complementarity of methods to embed outcome measures into the context. The context of active implementation and adjustments were examined qualitatively, using the core implementation components described by Fixsen et al. [30] as a framework: (1) staff selection; (2) preservice and in-service training; (3) ongoing coaching and consultation; (4) staff evaluation; (5) decision support data systems; (6) facilitative administrative support; and (7) systems interventions. We chose to focus on two quantitatively measured outcome concepts to account for both providers and users of child health care: (a) adoption which is interchangeably used with initial implementation and refers to “*the intention, initial decision, or action to try to employ an innovation*” ([32] p. 69) and (b) acceptability which refers to “*the perception among implementation stakeholders* (e.g. parents) *that a given treatment, service, practice, or innovation is agreeable, palatable, or satisfactory*” ([32] p. 67).

### Participants

Twenty-one CSHNs (all female) participated in the implementation pilot. All CSNHs having scheduled appointments among children aged 4 years were expected to participate based on their occupation in one of the two centers and did not volunteer for participation. In total, the centers cover a birth cohort of approximately 500 children. In addition, three CSHNs from center B having scheduled appointments with children aged 6 years (yearly n = 150) in three schools, were expected to participate. We collected no additional personal or professional information concerning CSHNs or children/parents.

In the project group, seven researchers representing medicine, nursing, psychology, and health economics participated along with head of services and two CSHNs.

### Tools and procedures

Working notes from researchers (by TW and EM), including feedback and support requests from CSHNs, and monthly project meeting memoranda (n = 14, authored by the project manager (EA)) were identified and sorted consecutively for case audit documentation. Documents included areas of improvement concerning (a) integration with routines and systems within the centers; (b) systems support; (c) reported experiences with the screening tools and reports; and (d) information needs within health centers, and by families.

User data from the online systems log were exported and analyzed. We assessed adoption by CSHNs by (a) the CSHN user rate of the system in relation to the number of nurses given education and access; (b) the rate of questionnaires distributed to parents of 4-year-old children in relation to the number of appointments as registered in the administrative journal in the two centers; (c) the rate of children for whom questionnaires were distributed to both parents; (d) and the rate of children for whom nurses registered the level of follow-up and weight/height. We assessed parental acceptability by the parental response rate measured as the rate of questionnaires responded to by at least one parent.

### Analysis

We screened documents and qualitatively analyzed the text with a deductive approach, using Fixsen et al.’s [30] seven core implementation components for coding. All elements of evaluation as well as adjustments were sorted according to this framework. Subsequently, we applied a narrative approach to describe how CSHNs adoption and/or parental acceptability could be enhanced by adjustments of core concepts, serving as drivers for active implementation.

In conducting statistical analyses of rates and fractions from the system logs representing adoption and acceptability we applied Excel in Microsoft Office 365 (Redmond, WA, USA).

## Results

### Adjustments of core implementation components

Adjustments of core implementation components were mainly based on *staff evaluation* and an overview is provided in Table 1. In order to facilitate feedback from staff, we increased researchers´ availability and worked to link evaluation with adjustments of the other components which are integrated and compensatory. First, CSHNs reported the usefulness of the tool in creating dialogue with families and found parents to be positive and more prepared during appointments. We hence emphasized this usefulness perspective in further *coaching and consultation*, of which one focus was repeatedly conveyed: CSHNs reported concerns about whether families with the greatest needs actually responded to the questionnaires. Specifically, they worried about how to reach parents who were not native Norwegians, as questionnaires were distributed in Norwegian only. We hence provided centers with several paper-based questionnaires in foreign languages (as requested from CHSNs based on their needs, e.g. Arabic) to support professional interpreters when needed.

**Table 1:** Integrated & compensatory adjustments of core implementation components [29] from the pilot implementation

	Staff selection	Preservice and in-service training	Ongoing coaching and consultation	Staff evaluation	Decision support data systems	Facilitative administrative support	Systems intervention
Staff evaluation	-Time-consuming for selected staff	-Difference in readiness and how fast CSHNs become familiar with tools -Specific user issues in online system	-Usefulness of tool to create dialogue -Concerns about nonresponders	-CSHNs needing to be involved by own premises	-Concerns about nonresponders -Difference in distribution of assessments to one vs. two parents -Concerns about CSHNs use	-Time-consuming for selected staff -CSHNs concerns of data protection	-Need for integration with electronic patient record -Double log-in
Adjustments	-Rearrange work tasks for medical secretary -Assistants to register children & guardians	-Extra visits in centers -Fewer CSHNs present to enhance individual support & feedback -Continuous improvement of online handbooks	-Emphasize usefulness in coaching -Provide paper-based questionnaires in foreign languages -Communicate adoption & acceptability	-Increase researchers´ availability	-Use systems log to assess parental acceptability -Use systems log to assess CSHNs adoption	-Assistants to register children & guardians -Information about data security protection by head of services	-Develop logical scheme for systems integration according to CSHNs´ working processes -Dialogue with system provides -Launching integration project

*Abbreviations: CSHN *child and school health nurse

The CSHNs reported that it was time-consuming to register children and their guardians in the system, and to distribute questionnaires in addition to scheduling appointments. Hence, we conducted additional *staff selection*. In center A, we rearranged work tasks for one medical secretary to support CSHNs with the distribution of questionnaires in advance of scheduled appointments, and this was also done from January 2020 in center B. Additionally, to *facilitate administrative support*, scientific assistants registered children and their guardians in the online system in advance of questionnaire distribution. Concerning *administrative support*, the CSHNs reported concerns about using a personal login ID instead of their professional ID. This concern was addressed by the head of services providing them with information about how data security protection was handled and assured. We sought support and acknowledgment from the head of services before allowing CSHNs to extend use of the online tool to cover 2- and 6-year-old appointments in addition to 4-year-old appointments.

CSHNs were different in how fast they became familiar with the online tool and readiness for extended use. We hence adjusted *preservice and in-service training* by paying extra visits in centers including fewer CSHNs at each visit to enhance individual support and feedback. We also improved online handbooks continuously, related to issues raised in support requests.

The CSHNs reported that integration between the electronic patient record and the online tool would have enhanced more seamless and effective working processes. During the entire pilot phase, the tool was used separately and without integration with electronic patient records. A main obstacle in daily work appeared to be the need to log into both the online tool and the electronic patient record. To overcome the lack of integration between the electronic patient record and the online tool, we developed a scheme for *systems interventions* defining each necessary integration point that was logically adapted to the working processes of CSHNs within the two systems. We initiated dialogue with system providers and launched a project for integrating the two systems.

From the online tool we utilized systems log as *decision support data systems*. We exported user rates from the system to assess CSHNs adoption as well as parental acceptability, used both for *ongoing coaching and consultation* of CSHNs and to meet current study aims reported in the following.

### Child and school health nurses´ adoption to the innovation

Of the 21 CSHNs educated and given access, 19 used the online tool giving an adoption rate among CSHNs of 90 %. Between October 1, 2019 and January 21, 2020, the online tool was used for nine appointments with 2-year-olds, 119 with 4-year-olds, and 59 with 6-year-olds. During the same period, 155 children were scheduled for 4-year-old appointments in the two centers. Overall adoption rate related to number of scheduled 4-year appointments was hence 77 %. As presented in Table 2, adoption rate was higher in center A than B (96 vs. 55 %).

**Table 2:** Child and school health nurses´ adoption to, and parental acceptability of the health service innovation at 4-year appointments in center A versus center B

	Center A	Center B
Scheduled appointments, n	82	73
Use of online assessments, n	79	40
Adoption rate, %	96 %	55 %
Distribution rate to two parents, n (%)	16 (20)	34 (85)
Parental response (acceptability) rate^a^, n (%)	63 (80)	34 (85)

^a^ Responses given by at least one parent of the child

### Parental acceptability of the innovation

At 4-year-old appointments, the overall response rate by at least one parent of the child reflecting parental acceptability was 97/119 (82 %). The user rate by CSHNs was 101/119 (85 %) registering the child’s height/weight data at the follow-up. As given in Table 2, CSHNs distributed questionnaires to two parents of the child more often in center B than in center A. The parental response rate reflecting acceptability was likewise higher in center B than in center A (Table 2).

In Table 3, parental acceptability represented by response rate are presented for 2- (center A) and 6-year (center B) appointments. Rate was higher in center B than A (98 vs. 78 %) along with higher rate of distribution to two parents (74 vs. 33 %). The CSHN user rate to register height/weight and level of follow-up were similar as parental response rate at 2-year appointments (78 %), and in accordance with parental response rate at 6-year appointments (95 %).

**Table 3:** Parental acceptability of the health service innovation at 2-year (center A) and 6-year appointments (center B)

	2-year appointments (*n* = 9)	6-year appointments (*n* = 59)
Distribution rate to two parents, n (%)	3 (33)	43 (74)
Parental response (acceptability) rate^a^, n (%)	7 (78)	58 (98)

^a^ Responses given by at least one parent of the child

## Discussion

Core implementation components were adjusted throughout the pilot implementation mainly based on staff evaluation. Reciprocity between staff evaluation and increased availability by researchers was central to integrate active implementation adjustments. The overall CSHNs adoption rate was satisfactory and higher in center A, where a medical secretary supported the nurses through the entire pilot phase, than in center B. Parental acceptability was overall high with highest response rates among parents of 6-year-old children compared with younger ones, and in cases where both parents received the questionnaires.

The systematic use of validated instruments in screening children’s development and health has been found to be more efficient than experience-based practice in identifying children at risk [16, 17]. Our results in terms of adoption and acceptability support the idea that it is possible to screen a high proportion of children, provided active implementation drivers is efficiently arranged and adjusted.

Despite the availability of screening instruments and national guidelines [22], nurses worldwide have previously reported the need for structured screening and improved decision support [15]. Without appropriate tools, they hesitate to react to children’s needs because of fear of damaging the professional CSHN–parent relationship [15, 18]. Staff evaluation in the current project has so far not revealed such challenges of balancing screening with support if the specific needs of a child were identified by the measures. The CSHNs reported positive experiences concerning preparedness for appointments, and that questionnaires provided positive opportunities to facilitate dialogue with parents. Such experiences were subsequently emphasized in implementation coaching for improved adoption of the innovation. The CSHNs experiences may also reflect parental acceptability, leading parents to meet more prepared from responding on child health assessments in advance of appointments. Our findings are supported by previous research showing that besides identifying children at risk, the use of clinical instruments in connection with health consultations might be effective in increasing discussion about emotional and psychosocial functioning [33]. Assessments may hence also strengthen the focus on clinical and measurable endpoints in preventive child healthcare, as pinpointed in previous research [2].

Although staff evaluation in the current project included reports on the utility of the instruments, it was also reported as time-consuming, which was accentuated by the lack of integration between data systems. Moreover, use of the online tool was initially new and unfamiliar to the CSHNs. However, once they had gained experience from using the tool in a couple of consultations, lack of familiarity was not an obstacle. Nevertheless, enhanced integration, including a single login, is warranted to support not only clinical decisions, but also time-efficient routines.

Besides the implementation of an online child health assessment tool, the project also changed clinical routines by integration of parent-reported measures and structured decision support. Even though structured screening is supported for identification, decision support, and evaluation in the literature [15–20], implementation of such routines and tools comes in addition to online systems implementation. New routines, tools, and systems might thus interact to both enhance and reduce nurses’ and parents’ adoption and acceptability, respectively. Routine outcome monitoring—for example, in mental health services—is considered important to individualize care and use resources effectively. The implementation of such systems in collaboration with, and training of clinicians is needed [19]. The relatively high adoption among CSHNs using the tool in the pilot implementation phase indicates that such collaboration was welcome and that it was possible to manage appropriately.

Parental acceptability was supported by high rates (77–98 %) of logins and responses by parents before appointments in the centers. Nevertheless, attention to nonresponders is important, as lack of response might reflect postponed or cancelled appointments, inability to login securely, lack of internet access, and/or language problems. CSHNs repeatedly shared concerns about nonresponders who might reflect families needing enhanced support from child health services. It is known that a pitfall of screening for intervention might be that deprived children and families with the greatest needs participate less in screening programs [34]. The consequences could be that children at risk are still not identified and appropriate intervention steps are not taken. Although more cases can be identified by the use of validated screening tools [20, 21], identification also depends on the response or acceptability rate and clinical adoption of the screening and/or surveillance systems. Nevertheless, the need to improve identification of vulnerable children at risk for appropriate intervention as early as possible is evident. Children may improve health and well-being based on improved social skills, improved parental mental health, and improved relational qualities within families [7]. Specifically, early interventions targeting parental reflective functioning and child–parent attachment are efficient [35], as well as treatments for anxiety [36]. However, the prevalence rates of anxiety triple treatment rates [37, 38]. Therefore, further challenges concerning nonresponders within the project as well as in programs implementing structured screening tools should not be ignored.

The current study inspired initiation of a project on systems integration, in which we also included The Norwegian Institute of Public Health as a partner and established dialogue with The Norwegian Directorate of E-health. Those institutions hold national aims and know-how concerning relevant health data needed about the child and adolescent population, and the process concerning one citizen–one patient record across health services, respectively. The `Starting Right´ health care innovation also complies with national guidelines for child and school health services [22] and hence have the potential to be transferred and applied nationally beyond the study context. The study partners, representing researchers from both primary and specialist health care, as well as higher education of health care staff, are also well equipped to strengthen use of evidence-based screening and patient reported outcome measures, and develop solutions that could be transferred and applied nationally and to related health care services.

### Strengths and limitations

This pilot implementation study was strengthened by the use of Fixsen et al.’s implementation framework for design and evaluation [30]. The comprehensive material, consisting of working notes and meeting memoranda during the implementation period, and the structured data from the online system log also strengthened the study. The pilot study was also limited and did not include all instruments we planned to implement or children younger than 2 or older than 6 years. Hence, experiences and results from full implementation and municipalities of different size might vary. We do not have any information concerning nonresponders among parents, or about CSHNs not adopting the system. However, the possibility of controlling the pilot implementation, and collaboration concerning this extensive and complicated change of tools and routines within the pilot phase was strengthened by limiting the pilot study to one municipality with a common head of services.

## Conclusions

Core implementation components were adjusted throughout the pilot implementation and informs further implementation of the ‘Starting Right’ health service innovation. The overall CSHNs adoption rate was satisfactory and higher where administrative support was provided. Parental acceptability, measured as the response rate, was high with a tendency for higher rates when both parents received the questionnaires, as well as for 6-year-old appointments compared with appointments for 4- and 2-year-old children.

Implications for practice could be to further emphasize the reciprocity between innovation and implementation developers and staff evaluation to adjust implementation drivers as well as the innovation systematically and continuously. More in-depth knowledge from qualitative interviews concerning experiences of both CSHNs and parents is warranted to further elaborate the `what´, `why´, and `how´ of practitioners´ adoption and parental acceptability. Such studies should integrate both experiences with the implementation, as well as experiences with the content of the innovation implemented. Further research on how systematic screening in preventive child healthcare may improve children´s health is also warranted.

## Data Availability

The datasets used and/or analyzed during the current study are not publicly available due to General Data Protection Regulation but are available from the corresponding author on reasonable request.

## Abbreviations

CSHN: child and school health nurse
SDQ: Strength & Difficulties Questionnaire
